# Sonographically Positive Fetal Heartbeat in Unilateral Tubal Twin Pregnancy as a Rare Case With Literature Review

**DOI:** 10.7759/cureus.39697

**Published:** 2023-05-30

**Authors:** Emine Öztürk, Hilal Aktürk

**Affiliations:** 1 Obstetrics and Gynaecology, Bakırköy Sadikonuk Education and Research Hospital, Istanbul, TUR; 2 Obstetrics and Gynaecology, Bakırköy Sadi Konuk Education and Research Hospital, Istanbul, TUR

**Keywords:** spontane, unilateral, twin, tubal pregnancy, ectopic pregnancy

## Abstract

Ectopic pregnancies occur when a fertilized egg implants outside the uterus, usually in the fallopian tube. Twin ectopic pregnancies are rare and pose significant diagnostic and management challenges. This case report presents the clinical details and management of a unilateral twin ectopic pregnancy in a 31-year-old female patient. The purpose of this report is to highlight the complexities associated with the diagnosis and management of this uncommon condition. In this case, we performed the left salpingectomy. We confirmed pathologically and histologically in pregnancy in the same tube.

## Introduction

Ectopic pregnancies account for approximately 1-2% of all pregnancies and can present with various clinical manifestations. We present a case of a unilateral twin ectopic pregnancy, where both embryos are implanted in the same fallopian tube. Same time, ectopic pregnancy is one of the gynecological emergencies. It is still considered one of the most important causes of maternal death among emergency pathologies [1-3]. This case report describes a sonographically positive fetal heartbeat in a unilateral tubal twin pregnancy, emphasizing the importance of early detection and appropriate management.

## Case presentation

**Figure 1:**
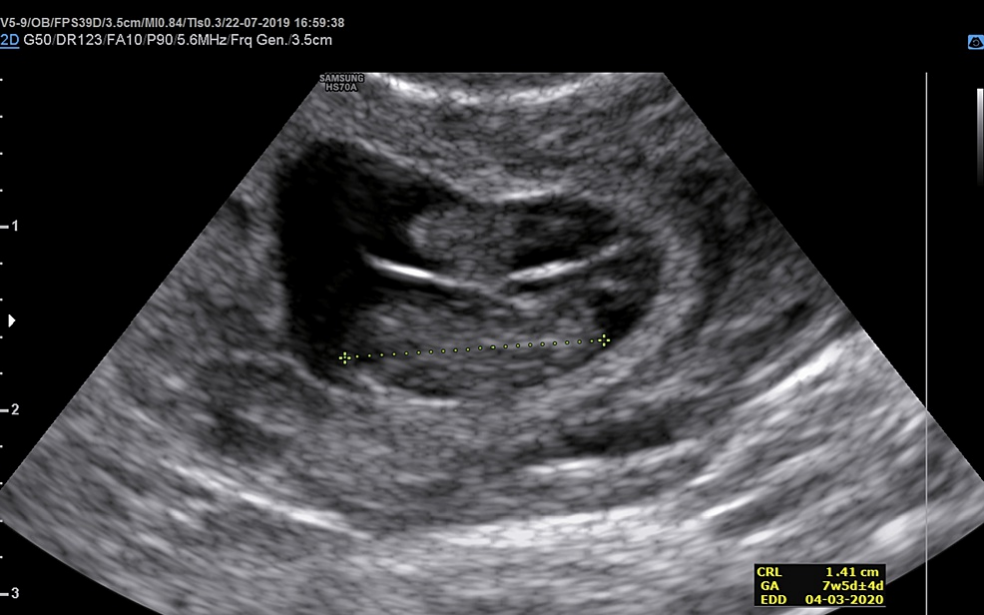
Transvaginal ultrasound showing twin live pregnancy was measured 11.2 mm and 11.4mm, which corresponds to the gestational age of 7 weeks 5 days and 7 weeks 4 days

A 31-year-old patient (gravida 4, para 3 c/s) was admitted to the clinic with acute abdominal pain and vaginal bleeding lasting four hours. The patient applied to the emergency service for the suspicion of left ectopic pregnancy in routine ultrasonography, whose gestational age was seven weeks and five days according to her last menstrual period. The patient was in good condition, conscious and well-oriented. Physical examination revealed abdominal tenderness and mild tenderness in the left adnexal region. Vaginal bleeding was mild. Ultrasonography revealed a thin endometrium and a complex mass of 32 x 42 mm adjacent to the left ovary, which is two thick-walled, fluid-filled cystic masses of 13 and 15 mm in size with further investigations. Additionally, both gestational sac containing a fetal pole with a sonographically positive heartbeat was identified within the left fallopian tube (Figure 1). Endometrial thickness was 12 mm, and minimal fluid was detected in the Douglas pouch. Her laboratory findings were the following: hemoglobin, 11,2 g/dl; White blood cell 21,120 /ul; serum βhCG level 25,696 mIU/ml. In light of all the findings, the patient was diagnosed with left unilateral twin tubal pregnancy.

**Figure 2:**
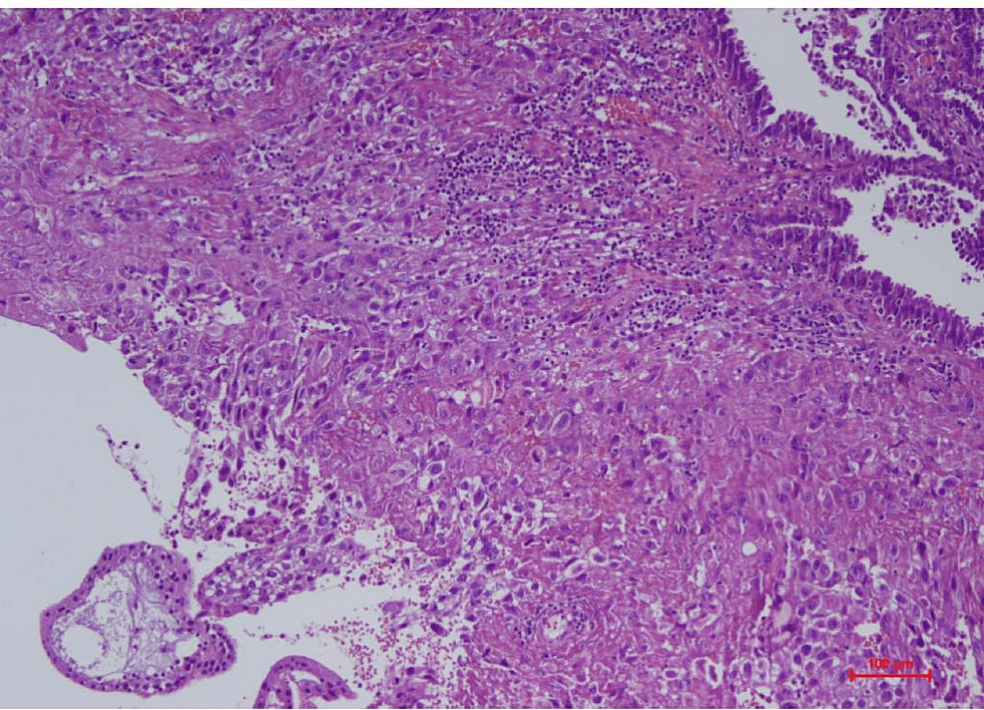
Tubal Pathology Sampling with Hematoxylin Eosin

We immediately terminated the pregnancy by laparoscopic unilateral left salpingectomy under general anesthesia. Our surgical findings detected a 5 cm ectopic mass in the ampulla region. The postoperative period passed without complications. The patient was discharged uneventfully after a postoperative follow-up. Pathologically and histologically demonstrated a twin pregnancy in the same tube and Aries-Stella phenomenon detected in an endometrial cavity (Figure 2).

## Discussion

Although twin ectopic pregnancy is quite rare, its incidence is increasing. The literature has reported 299 cases between 1911 and 2023, and this number has been determined in the last 15 years [4]. Assisted reproductive endocrinology is the biggest reason for the increase in twin tubal pregnancies. Spontaneous twin ectopic pregnancy is extremely rare and is detected in one in every 125,000 pregnancies [5]. In 1994, transvaginal ultrasonography detected a tubal pregnancy accompanied by twin heartbeats [6]. As far as is known, there are only 13 sonographically positive fetal heartbeats in unilateral tubal twin pregnancy. Cases of cardiac activity have been reported in the literature [3,5-14]. Chang et al. recently reported the 12th twin unilateral tubal pregnancy, followed by Chen et al., who reported the 13th twin unilateral tubal pregnancy. Therefore, our case represents the fourteenth instance reported in the existing literature. In four cases, there were no risk factors such as tubal pathology or surgery, and in only one case surgery was not performed (Table 1).

**Table 1:** Recent Cases for Unilateral Twin Tubal Pregnancy Presenting Fetal Cardiac Activity

Authors	Year	Risk Factors	Clinic Feature	Treatment
Gualandi et al. [6]	1994	None	Cardiac activity(+)	Laparotomy
Eddib et al. [11]	2006	None	Cardiac activity(+) Ruptured	Laparoscopy
Karadeniz et al. [12]	2008	İnf treatment, smoke	Cardiac activity(+) Unruptured	Metothrexat
Karanjgoober et al. [8]	2009	Retained product of conception	Cardiac activity(+) Ruptured	Laparoscopy
Langoria et al. [3]	2014	Previous Tubal Surgery	Cardiac activity(+) Unruptured	Laparoscopy
Ghanbarzadeh et al. [13]	2015	Previous Tubal Surgery	Cardiac activity(+) Ruptured	Laparotomy
Chang Kim et al. [5]	2018	None	Cardiac activity(+) Unruptured	Laparoscopy
Chen-June et al. [14]	2019	None	Cardiac activity(+) Unruptured	Laparoscopy
Martin et al. [7]	2021	None	Cardiac activity(+) Unruptured	Laparoscopy

The best treatment option involves various factors; hemodynamic stability, medical conditions, presence of a heartbeat, and fertility desire should be investigated. Systemic methotrexate is successfully administered to a hemodynamically stable patient with a beta-hCG value of less than 5000 mIU/ml and no fetal heartbeat [10]. However, this is not suitable for twin ectopic pregnancies. In the 40-case study by De Los Rios, systemic methotrexate was administered in only one case [1]. In our case, the patient was counseled about the potential risks of a twin ectopic pregnancy, including tubal rupture and hemorrhage. After obtaining informed consent, the patient underwent a laparoscopic left salpingectomy, which involved tube removal. The procedure was successful, and the patient had an uneventful recovery.

In cases of acute ruptured ectopic pregnancy, hemodynamically unstable patients, failed medical management, or those who are contraindicated for medical management, surgical management is preferred. Laparoscopic management is becoming more popular due to its lower cost, faster recovery, shorter operation time, and hospital stay. However, salpingectomy is still the recommended treatment. In cases where fertility preservation is desired, salpingostomy may be considered. For unilateral tubal twin pregnancies, surgical management is typically the preferred treatment, as supported by the literature, similar to singleton ectopic pregnancies [15].

The morbidity and mortality of ectopic pregnancy have decreased with improved diagnosis and treatment protocols. It has a life-threatening potential in the first three months of life. It is known to be responsible for 9%-13% of pregnancy-related deaths [16].

## Conclusions

Unilateral twin ectopic pregnancies are exceptionally rare, and their diagnosis and management can be challenging. Early recognition and prompt intervention are crucial in preventing complications such as tubal rupture and hemorrhage. This case report highlights the importance of maintaining a high index of suspicion in patients with an acute abdomen and the need for close monitoring and timely surgical intervention. Further research and clinical reports are warranted to improve our understanding of this uncommon condition and optimize its management.
